# Treatment of Peritoneal Carcinomatosis by Targeted Delivery of the Radio-Labeled Tumor Homing Peptide ^213^Bi-DTPA-[F3]_2_ into the Nucleus of Tumor Cells

**DOI:** 10.1371/journal.pone.0005715

**Published:** 2009-05-27

**Authors:** Enken Drecoll, Florian C. Gaertner, Matthias Miederer, Birgit Blechert, Mario Vallon, Jan M. Müller, Andrea Alke, Christof Seidl, Frank Bruchertseifer, Alfred Morgenstern, Reingard Senekowitsch-Schmidtke, Markus Essler

**Affiliations:** 1 Department of Nuclear Medicine, Klinikum-rechts-der-Isar, München, Germany; 2 European Commission, Joint Research Centre, Institute for Transuranium Elements, Karlsruhe, Germany; National Cancer Institute, United States of America

## Abstract

**Background:**

α-particle emitting isotopes are effective novel tools in cancer therapy, but targeted delivery into tumors is a prerequisite of their application to avoid toxic side effects. Peritoneal carcinomatosis is a widespread dissemination of tumors throughout the peritoneal cavity. As peritoneal carcinomatosis is fatal in most cases, novel therapies are needed. F3 is a tumor homing peptide which is internalized into the nucleus of tumor cells upon binding to nucleolin on the cell surface. Therefore, F3 may be an appropriate carrier for α-particle emitting isotopes facilitating selective tumor therapies.

**Principal Findings:**

A dimer of the vascular tumor homing peptide F3 was chemically coupled to the α-emitter ^213^Bi (^213^Bi-DTPA-[F3]_2_). We found ^213^Bi-DTPA-[F3]_2_ to accumulate in the nucleus of tumor cells in vitro and in intraperitoneally growing tumors in vivo. To study the anti-tumor activity of ^213^Bi-DTPA-[F3]_2_ we treated mice bearing intraperitoneally growing xenograft tumors with ^213^Bi-DTPA-[F3]_2_. In a tumor prevention study between the days 4–14 after inoculation of tumor cells 6×1.85 MBq (50 µCi) of ^213^Bi-DTPA-[F3]_2_ were injected. In a tumor reduction study between the days 16–26 after inoculation of tumor cells 6×1.85 MBq of ^213^Bi-DTPA-[F3]_2_ were injected. The survival time of the animals was increased from 51 to 93.5 days in the prevention study and from 57 days to 78 days in the tumor reduction study. No toxicity of the treatment was observed. In bio-distribution studies we found ^213^Bi-DTPA-[F3]_2_ to accumulate in tumors but only low activities were found in control organs except for the kidneys, where ^213^Bi-DTPA-[F3]_2_ is found due to renal excretion.

**Conclusions/Significance:**

In conclusion we report that ^213^Bi-DTPA-[F3]_2_ is a novel tool for the targeted delivery of α-emitters into the nucleus of tumor cells that effectively controls peritoneal carcinomatosis in preclinical models and may also be useful in oncology.

## Introduction

A single, high linear energy transfer α-particle can kill a tumor cell [1]. Therefore, α-particle emitting isotopes may emerge as effective novel tools in cancer therapy [2]. Due to its high linear energy transfer, short half life and uncomplicated use ^213^Bi is promising for medical applications. To avoid toxic side effects, targeted delivery of such isotopes into tumors is a prerequisite of their application in oncology. α-particles kill cells due to induction of double strand brakes in DNA with a high relative biological effectiveness. As the range of the particles is only 28–100 µm in mammalian tissues, the development of carriers mediating specific uptake into the nucleus of tumor- or tumor endothelial cells is important to optimize therapeutic efficacy. Therefore, an ideal carrier for radionuclide therapy with α-emitters would mediate the specific uptake of the radionuclide selectively into the nuclei of tumor cells but not into cells in normal organs. Considering the short physical half life of ^213^Bi (45.6 minutes) a quick internalization of the carrier is essential. Tumor-specific monoclonal antibodies coupled to α-emitters such as ^225^Ac, ^211^At, ^149^Tb or to ^213^Bi have already been successfully tested by several groups in a number of clinical and preclinical studies in different tumor entities including leukemia, glioma, melanoma, lymphoma, breast, ovarian, neuroblastoma as well as colon-and prostate cancer [1], [3], [4], [5], [6], [7]. Peptides may also be used as conjugates for the targeted delivery of α-emitters into tumors and may be a promising option for cancer therapy [8]. Although it has been shown that in animal models ^225^Ac and ^213^Bi coupled to DOTATOC are effective in the treatment of neuroendocrine tumors, there is only limited experience with alpha particle emitting radio-peptide-conjugates in the therapy of cancer [9], [10]. As a first step to develop radionuclide therapies using tumor homing peptides, it is important to test the binding affinity, stability and therapeutic efficacy of alpha-emitters coupled to such peptides *in vitro* and *in vivo*.

Peptide libraries displayed on phage can be screened in vivo for phage particles that home to a specific target [11]. A number of peptides capable of homing to tumor vasculature were isolated in this manner [12]. In preclinical models, targeted drug delivery into tumors has been achieved by conjugation of anti-cancer drugs with vascular tumor homing peptides [13]. F3 is a 32 amino acid vascular tumor homing peptide that binds specifically to nucleolin on the surface of tumor cells [14], [15]. In resting cells, nucleolin is primarily expressed in the nucleus. In contrast, in proliferating tumor or tumor endothelial cells, nucleolin is cyclically transported from the nucleus to the cell surface and back by a specific shuttle mechanism [16]. Therefore, nucleolin is potentially an appropriate target for selective tumor therapies. Indeed, it has been reported that F3 is capable of transporting nano-particles or fluorescent dyes such as FITC into the nuclei of tumor cells and tumor endothelial cells *in vitro* and *in vivo* [17].

Peritoneal carcinomatosis is a widespread dissemination and implantation of tumors throughout the peritoneal cavity. It is a frequent and serious complication of intra-abdominal cancers of the gastrointestinal tract, including carcinomas of the colon, pancreas and stomach as well as ovarian cancer [18]. Peritoneal carcinomatosis also occurs in patients with primarily extra-abdominal tumors such as breast cancer and melanoma. The majority of patients who are diagnosed with ovarian or pancreatic cancer already have peritoneal disease at the time of diagnosis. The vague signs and symptoms associated with peritoneal disease, such as bloating and early satiety, often delay the diagnosis and the initiation of treatment, leading to a poor prognosis [19]. Additionally, the lack of an effective treatment strategy leaves patients at significant risk of recurrence. Therefore, there is an urgent need to develop more effective tools for the treatment of peritoneal carcinomatosis. Recent evidence suggests that radionuclide therapy may be more beneficial than intra-peritoneal chemotherapy as treatment for peritoneal carcinomatosis [7].

We generated an F3 dimer coupled to DTPA which chelates ^213^Bi and tested binding affinity, bio-distribution and anti-tumor activity of this construct (^213^Bi-DTPA-[F3]_2_) *in vitro* and *in vivo* in a pre-clinical model of peritoneal carcinomatosis. We found that ^213^Bi-DTPA-[F3]_2_ binds with high affinity to tumor cells, is internalized into the nuclei of tumor cells *in vitro* and is effective in treatment of peritoneal carcinomatosis.

## Materials and Methods

### Ethics Statement

All experiments were performed according to the German law for the protection of animals. The animal experiments were approved and supervised by the Government of Upper Bavaria (Regierung von Oberbayern) (record number 55.2-1-54-2531-34-02 and 55.2-1-54-2531-52-07).

### Materials

All materials not further specified were purchased from Sigma, Deisenhofen, Germany.

### Cell Culture

The tumor cell lines MDA-MB-435 (human breast cancer), MIAPACA (human pancreas carcinoma), OVCAR 3 (ovarian cancer) and CMT 93 (murine colon cancer) were grown in RPMI 1640 Medium (Biochrom, Berlin, Germany) supplemented with 1% penicillin/streptomycin (10.000 U/10 mg/ml, Biochrom), 1% gentamycine, 1% L-glutamine (200 mM, Biochrom) und 10% fetal calf serum (FCS, Biochrom). EMT6-cells were grown in Waymouth Medium (Gibco) supplemented with 10% FCS und penicillin/streptomycin (Gibco, 0.1 mg/ml).

### Synthesis and labeling of ^213^Bi-DTPA-[F3]_2_ and ^68^Ga-DTPA-[F3]_2_

DTPA-[F3]_2_ was synthesized by GenScript Corporation, Piscataway, NJ. In this construct the chelator DTPA is coupled to F3 via its N-terminus. ^213^Bi was eluted from a ^225^Ac/^213^Bi in-house-generator provided by the Institute of Transuranium Elements [20], [21] using elution buffer (0.1 M HCl, 0.1 M NaI, pH 5). For coupling of ^213^Bi to DTPA-[F3]_2_ 3.3 µg DTPA-[F3]_2_/mCi ^213^Bi were incubated with 100 µl ascorbic acid (40 mg/ml) und 3 M NH_4_Ac (pH 5.3) at room temperature for 10 minutes. Labelling efficacy was tested by ITLC and was >90% in all experiments. To test stability the constructs were diluted 1∶10 with murine serum, cell culture media or ascites from mice with peritoneal carcinomatosis and incubated at 37°C for the indicated times. 1–2 µl of the solution were then submitted to ITLC. The amount of activity bound to the peptide was then determined using a γ-counter.

### Internalization of ^213^Bi-DTPA-[F3]_2_ and cell fractionation in vitro

MDA-MB-435 cells were grown to a density of 1×10^7^ cells/dish in 10 mm culture dishes. For fractionation of cultured MDA-MB-435 cells after incubation with 1.85 MBq (33 ng/ml) ^213^Bi-DTPA-[F3]_2_ for 5, 10, 20, 45, and 90 minutes, cells were washed three times with ice cold PBS. Culture dishes were placed on ice and 4 ml of ice cold Nuclei EZ lysis buffer (Nuclei Isolation Kit from Sigma, Deisenhofen, Germany) were added. Cells were harvested using a bladed cell scraper. The entire cell lysate from each plate was transferred into a 15 ml centrifuge tube (Falcon), vortexed and set on ice for five minutes. The nuclei were then pelleted by centrifugation at 500×g for five minutes, washed by three times with EZ lysis buffer and resuspended in nuclei EZ storage buffer. The supernatant contains the cytoplasm and membrane fraction. The integrity of the nuclei was checked by microscopy. To determine the ^213^Bi-radioactivity concentration within the nuclei of tumor cells we determined the volume and the number of nuclei using a haemocytometer. The radioactivity present was determined by a using a γ-counter and used for calculation of the radioactivity concentration (MBq/µl).

### Cell viability test

1×10^6^ MDA-MB-435 cells were cultivated in 6 well plates. After 4 hours for cell adherence, the cells were treated with different activities of ^213^Bi-DTPA-[F3]_2_. Untreated cells and cells treated with free ^213^Bi were used as control. After 3 days, cells were detached with trypsin/EDTA and washed once with PBS. 50 µl of the cell solution were mixed with 0.4% trypan blue solution 1∶1 for 10 minutes. Total cell count and the rate of unviable cells were determined using a haematocytometer.

### Blocking of ^213^Bi-DTPA-[F3]2 binding to tumor cells

EMT-6 cells were grown to confluence on glass cover slips. Cells were washed with PBS and incubated with 40 ng/ml ^213^Bi-DTPA-[F3]_2_ in presence or absence of 400 ng/ml DTPA-[F3]_2_ for 30 minutes at room temperature. Cover slips were the washed by three times with PBS and analyzed with a micro-imager (µImager™, Biospace, Germany).

### Soft agar assay for colony formation

1×10^5^ MDA-MB-435, MIAPACA, OvCAR3 and CMT 93 cells were cultivated in 6 well plates for 24 h. Cells were treated with different activities of ^213^Bi-DTPA-[F3]_2_ in cell culture media (RPMI 1640). After 12 h cells were detached with trypsin, washed 3 times and resuspended in RPMI 1640. 200 µl cell culture medium with 4×10^4^ tumor cells were filled into a 15 ml Falcon tube and mixed with 0.7% low melting agarose and 3 ml 2× RPMI 1640. 1.5 ml of this suspension were added per well of 6-well plates covered with base agar. Cell culture dishes were then incubated at 37°C for one week and stained with 0.5 ml 0.005% Cresyl-violet-solution for the minimum of 1 hour. Colonies were counted using an inverted microscope (Zeiss, Oberkochen, Germany).

### Tumor model of peritoneal carcinomatosis

To generate intraperitoneal xenograft tumors, 6–8 week old SCID-mice (Charles River, Sulzfeld, Germany) were injected i.p. with 1×10^7^ MDA-MB435 tumor cells. Mice were weighed weekly and were sacrificed when the mean normal body weigth decreased <25% or when mice developped tumor side effects like ascites or reduced general condititon. All experiments were performed according to the German law for the protection of animals.

### Biodistribution of ^213^Bi-DTPA-[F3]_2_

^213^Bi-DTPA-[F3]_2_ (3.7 MBq) was injected i.p. into SCID-mice with intra-peritoneal growing tumors in an advanced stage 20 days after inoculation of tumor cells. After 45 minutes, mice were sacrificed, the tumors and the organs were surgically removed and the radioactivity present in each organ (percent of the injected activity/g = % ID/g), the tumors and the blood was measured with a γ-counter.

### Autoradiography and histology

Tumor bearing mice were injected i. p. with 3.7 MBq ^213^Bi-DTPA-[F3]_2_. The tumors and control organs were resected, snap frozen with liquid nitrogen and cut in 10 µm sections using a microtome (HM 500 O, Microm, Germany). Sections were then exposed to a micro-imager (µImager™, Biospace, Germany). For H&E staining 7.5 µm sections were cut, fixed with 37% formalin and stained with haematoxiline and eosine.

### PET-imaging using ^68^Ga-DTPA-[F3]_2_

7.4 MBq ^68^Ga-DTPA-[F3]_2_ in 100 µl PBS were injected i.p. into mice bearing intra-peritoneal xenograft tumors 20 days after inoculation. After 60 minutes mice were anesthetized (3% isoflurane, O_2_ 4 l/min) and examined with a small animal PET-scanner (MicroPET Focus 120, Siemens, Germany). Picture analysis was performed using Osirix™ software.

### Therapy of intraperitoneal xenograft tumors

SCID-mice (Charles River, Sulzfeld, Germany) were injected i.p. with 1×10^7^ MDA-MB-435 cells expressing firefly luciferase in 100 µl PBS. In the tumor prevention study groups of 8 mice were injected every second day with 1.85 MBq ^213^Bi-DTPA-[F3]_2_, 1.85 MBq ^213^Bi-DTPA or 100 µl PBS between day 4 and 14 after inoculation of the tumor cells. In the tumor reduction study groups of 8 mice were injected i.p. with 1.85 MBq ^213^Bi-DTPA-[F3]_2_ , 1.85 MBq ^213^Bi-DTPA or 100 µl PBS between the days 16 and 26 after inoculation of the tumor cells. All experiments were performed three times. The animals were controlled daily until they were sacrificed.

### Optical imaging

For optical imaging of xenograft tumors, animals were anesthetized by i.p. injection of 10 µl/g anesthesia solution (10% Ketavet/Pharmacia & Upjohn, Erlangen, Germany, 8% Rompun, Bayer, Leverkusen, Germany and 82% NaCl 0.9%). Simultaneously, 300 µl D-Luciferin (15 mg/ml, Synchem, Germany) were injected i.p. Imaging was performed 10 minutes p.i. using a cooled CCD-camera (Hamamatsu, Herrsching, Germany).

### Macroscopic reduction of cell clones in vivo

Two SCID-mice with intraperitoneal MDA-MB-435 xenografts were treated with 6×1.85 MBq ^213^Bi-DTPA-[F3]_2_ between the days 4 and 14 after inoculation of tumor cells as described above, two others with 6×100 µl PBS. 50 days after injection of xenografts, mice were sacrificed and the small intestine and the colon were preparated for macroscopic examination of tumor spread.

### Statistical Analysis

Statistical analysis of cell-survival-studies was performed using the student t-test for paired values and with t-test according to Lord and Moore for unpaired values. Kaplan-Meier curves were analyzed by a Log Rank Test.

## Results

### Binding and internalization of ^213^Bi-DTPA-[F3]_2_

**Figure 1:**
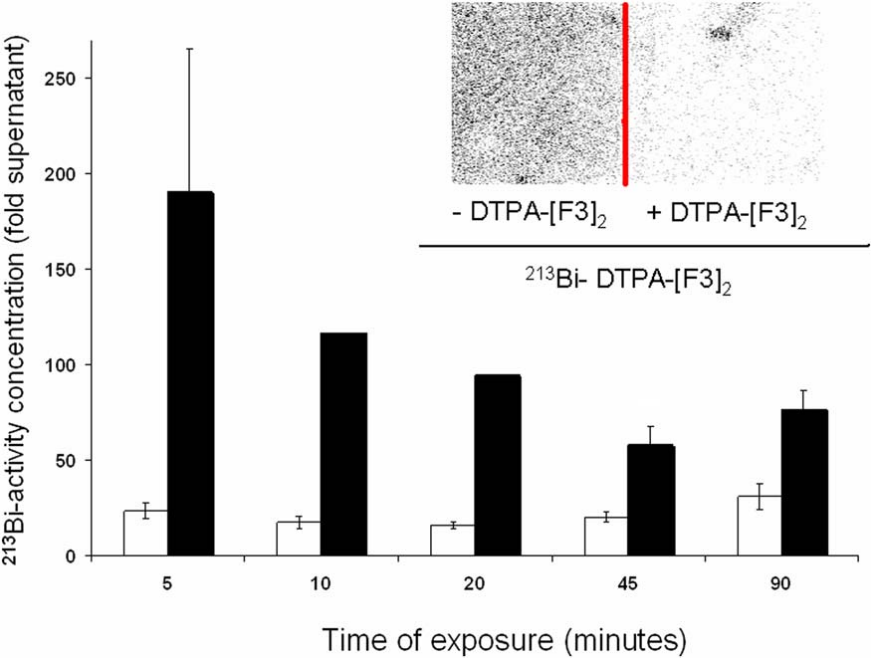
Internalization of ^213^Bi-DTPA-[F3]_2_ into MDA-MB-435 tumor cells. The radioactivity concentration present in the cytoplasm and the nuclei of cells incubated with 3.7 kBq/ml ^213^Bi-DTPA-[F3]_2_ for the indicated time periods was measured and compared to the radioactivity concentration in the supernatant. The bars represent the radioactivity in the nuclei (black, p<0.01, * = p<0.02) or the cytoplasm (white, p<0.01) compared to the supernatant. Values±SEM are shown. The inset shows binding of ^213^Bi-DTPA-[F3]_2_ to tumor cells grown on glass cover slips in presence or absence of unlabelled DTPA-[F3]_2_, visualized by a micro imager.

To couple F3 to the α-particle emitting isotope ^213^Bi, DTPA-[F3]_2_ was synthesized which is a F3-dimer linked to DTPA. The chelator DTPA effectively binds metal ions such as ^213^Bi. It was reported recently by Porkka et al. reported [14] that F3 is internalized into the nucleus of tumor cells and tumor endothelial cells *in vitro* and *in vivo*. We therefore asked whether also ^213^Bi-DTPA-[F3]_2_ would also be internalized into the nucleus of tumor cells. To address this question we incubated MDA-MB-435 tumor cells with ^213^Bi-DTPA-[F3]_2_ *in vitro* and after the time periods indicated in figure 1, separated the cytoplasm and the nuclei of the tumor cells and measured the radioactivity concentration present in each fraction. As shown in figure 1 activities measured per volume were up to 200fold higher in the nuclei and up to 30fold higher in the cytoplasm compared to the cell culture supernatant containing unbound ^213^Bi-DTPA-[F3]_2_, indicating that ^213^Bi-DTPA-[F3]_2_ accumulates in tumor cells. Peak activities were found as early as 5 minutes after initiation of exposure, but elevated levels were found for at least 90 minutes, i.e. two half-lifes of ^213^Bi. To test whether the uptake of ^213^Bi-DTPA-[F3]_2_ into tumor cells is specific we performed blocking experiments using DTPA-[F3]_2_ not labelled with ^213^Bi. Cells were grown on glass cover slips and incubated with ^213^Bi-DTPA-[F3]_2_±DTPA-[F3]_2_ for 5 minutes. After extensive washing the cover-slips were analyzed by a micro-imager to visualize radioactivity. The inset to figure 1 shows that binding of ^213^Bi-DTPA-[F3]_2_ to tumor cells can be blocked by an excess of unlabelled peptide consistent with specificity of binding.

### Anti-tumor activity of ^213^Bi-DTPA-[F3]_2_ in vitro

**Figure 2:**
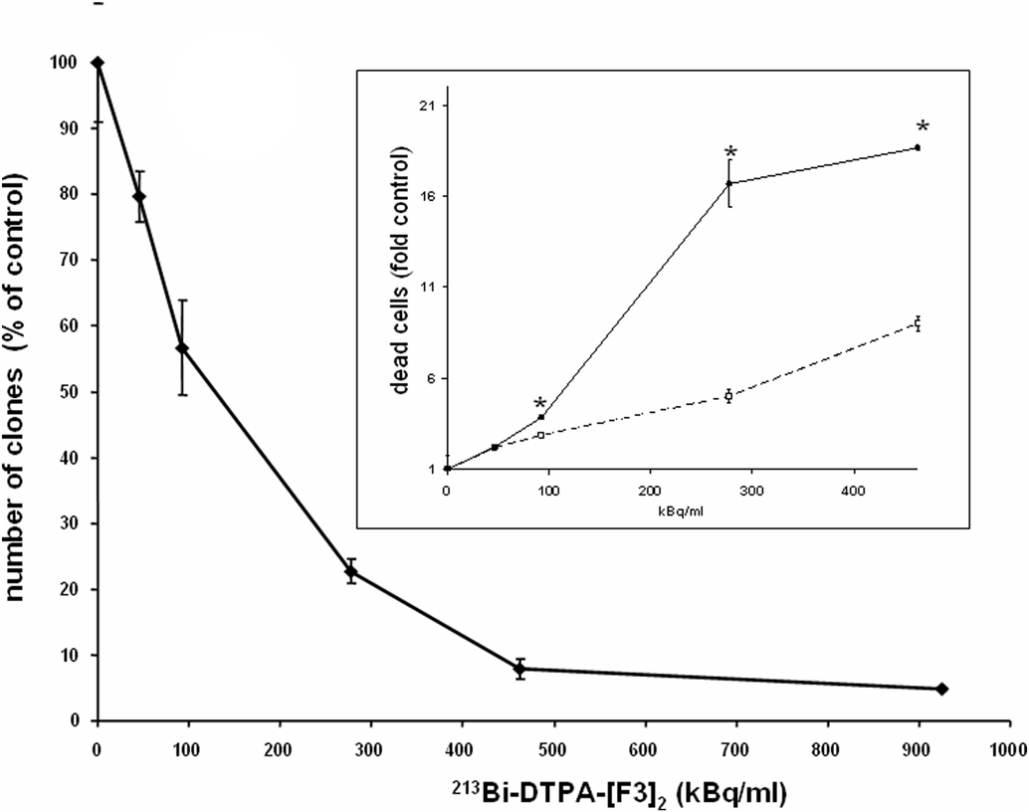
Anti-tumor-activity of ^213^Bi-DTPA-[F3]_2_ in vitro. MDA-MB-435 tumor cells were incubated for 12 h with different activities of ^213^Bi-DTPA-[F3]_2_ as indicated. The number of malignant cell clones growing in soft agar was determined after 14 days and compared to untreated cells. Values±SEM are shown (n = 6, p<0.01). The inset shows that cell death is induced by ^213^Bi-DTPA-[F3]_2_, as detected by Trypan blue staining of cells (solid line). ^213^Bi induced significantly lower numbers of dead cells (dashed line) (n = 3, * = p<0.01).

**Figure 3:**
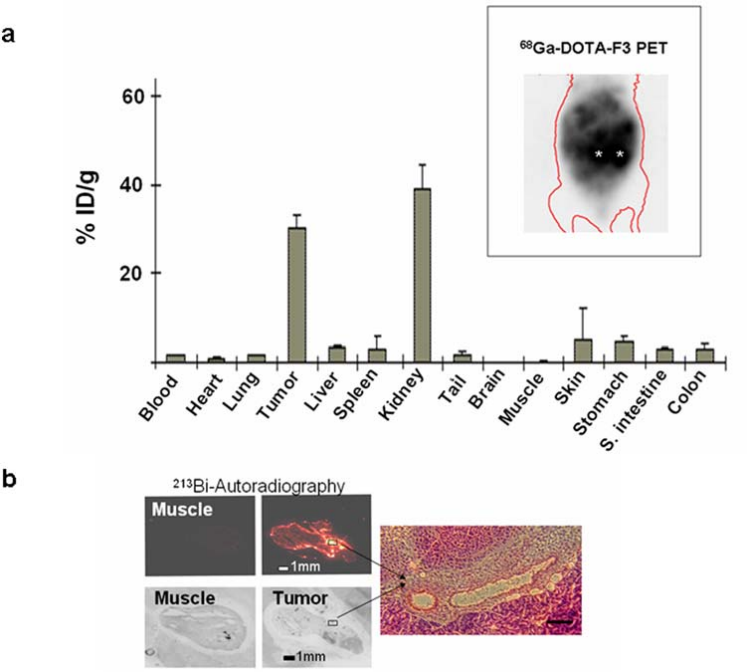
(a) Biodistribution of ^213^Bi-DTPA-[F3]_2_. 3.7 MBq of ^213^Bi-DTPA-[F3]_2_ were injected i.p. into mice bearing intra-peritoneal MDA-MB-435 xenograft tumors. After 45 minutes the ^213^Bi-DTPA-[F3]_2_ activity present in individual organs, the tumors and the blood was measured. Values represent the percentage of the injected dose/g tissue (%ID/g)±SEM. The inset depicts a mouse with peritoneal carcinomatosis imaged with ^68^Ga-DOTA-F3-PET. The asterics (*) indicates the location of the kidneys. b) Autoradiography studies were performed using histological sections of MDA-MB-435 xenograft tumors or muscle tissue. ^213^Bi-DTPA-[F3]_2_ was found in tumors 45 minutes after i. p. injection. ^213^Bi-DTPA-[F3]_2_ was found in the periphery of the tumor as well as in spots within the tumor tissue. H&E-staining and microscopy (100fold magnification) of the sections revealed that the intra-tumoral accumulation of ^213^Bi-DTPA-[F3]_2_ occurs in the perivascular region. The H&E-stained picture represents the area within the white box in the autoradiography picture. Negligible activities were found in autoradiography pictures of control organs such as muscle.

As F3 was shown to be internalized into the nucleus of tumor cells and therefore may be an appropriate carrier for α-particle emitting isotopes, we investigated the potential of ^213^Bi-DTPA-[F3]_2_ to effectively reduce the number of cells in a tumor cell population. For this purpose we performed colony formation assays (Figure 2). We found that ^213^Bi-DTPA-[F3]_2_ significantly reduced the number of clones resulting from MDA-MB-435 cells in a dose dependent manner (p<0.01). Furthermore, ^213^Bi-DTPA-[F3]_2_ was found to be effective on a variety of tumor cells from different tumor types frequently causing peritoneal carcinomatosis including pancreas carcinoma (MIAPACA), colon carcinoma (CMT 93) and ovarian cancer (OvCAR3). Table 1 indicates the ID50 values for these cell lines, i.e. the ^213^Bi-activity concentration reducing the number of malignant clones by 50% compared to a control. We also tested the cytotoxic effect of ^213^Bi-DTPA-[F3]_2_ by staining dead cells. The inset to figure 3 indicates that ^213^Bi-DTPA-[F3]_2_ induced cell death in a dose dependent manner. In contrast, free ^213^Bi is less active. These findings show that ^213^Bi-DTPA-[F3]_2_ has a specific anti-tumor activity *in vitro*. DTPA-[F3]_2_ not labelled with ^213^Bi did not block cell growth and did not induce cell death (10 pg/ml to 100 ng/ml; data not shown).

**Table 1:** The table shows the ID50 values of ^213^Bi-DTPA-[F3]_2_, i.e. the activity concentration reducing the number of clones in a colony formation assay by 50% in different cell lines (EMT6, CMT93, MIAPACA, OVCAR3, MDA-MD-435).

Cell Line	Organ	^213^Bi-DTPA-[F3]_2_ ID50 (kBq/ml)
EMT6	Breast Cancer	23.9
MIAPACA	Pancreas Carcinoma	32.3
CMT93	Colon Carcinoma	99.5
OVCAR3	Ovarian Carcinoma	94.0
MDA-MB-435	Breast Cancer	119

### Biodistribution of ^213^Bi-DTPA-[F3]_2_

We next asked whether ^213^Bi-DTPA-[F3]_2_ does accumulate in tumor cells in vivo. ^213^Bi-DTPA-[F3]_2_ (3.7 MBq) was injected i.p. into mice with intra-peritoneal xenograft tumors. After one half life of ^213^Bi, mice were sacrificed, the tumors and the organs were surgically removed and the radioactivity present in each organ, the tumors and the blood was measured with a γ-counter. Up to 32% of the injected dose/g was found in the tumors. No significant accumulation of ^213^Bi-DTPA-[F3]_2_ could be detected in all other organs except for the kidneys. The renal accumulation is most likely due to excretion of the radio-labeled peptide. The tumor/blood ratio was 17.6∶1 (Figure 3a). The tumor/intestine ration was 10.37∶1. To further demonstrate accumulation of F3 in intraperitoneal tumors we performed PET studies. Mice bearing intraperitoneal xenograft tumors were imaged 2 hours after injection with ^68^Ga-DOTA-F3 using a dedicated small animal PET machine. The inset to figure 3a depicts a representative PET-image. ^213^Bi-DTPA-[F3]_2_ may therefore be an appropriate tool for the therapy of peritoneal carcinomatosis.

To test in which tumor regions ^213^Bi-DTPA-[F3]_2_ accumulates within xenograft tumors autoradiography studies were performed. We found that ^213^Bi-DTPA-[F3]_2_-activity accumulates primarily in the periphery of the tumor and in peri-vascular regions. In contrast, only low amounts of ^213^Bi-activity were found in control organs such as muscle (Figure 3b).

### Treatment of peritoneal carcinomatosis by ^213^Bi-DTPA-[F3]_2_ in vivo

**Figure 4:**
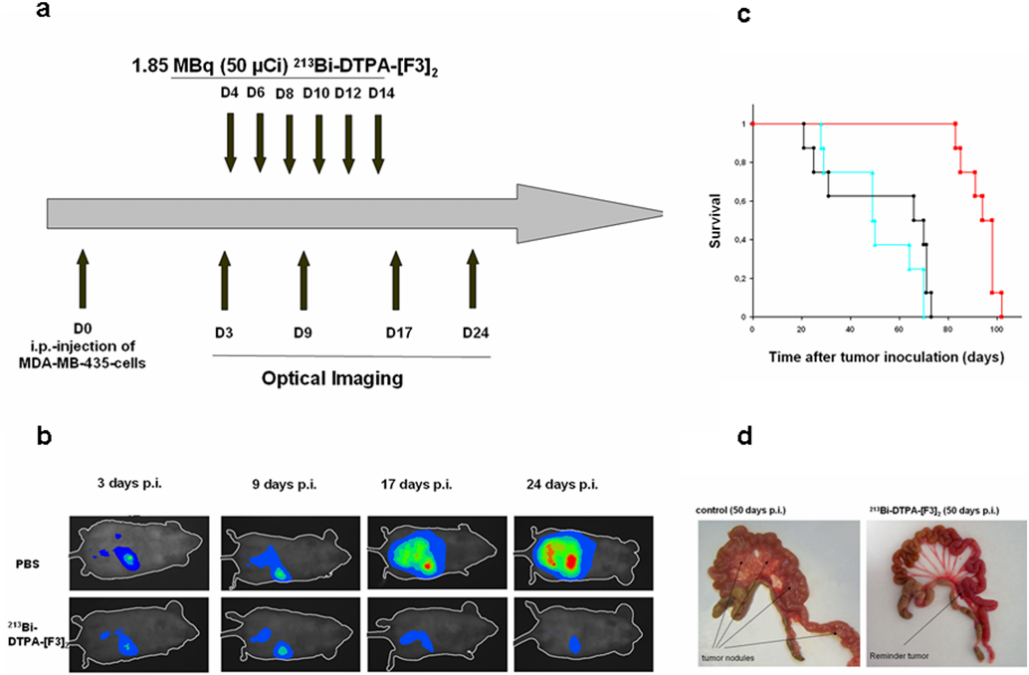
Tumor prevention study. (a) The scheme depicts the time points when ^213^Bi-DTPA-[F3]_2_, ^213^Bi-DTPA or PBS were injected and the time points when optical imaging was performed. (b) Assessment of the tumor growth of MDA-MB-435luc2 xenograft tumors by optical imaging in a mouse treated with ^213^Bi-DTPA-[F3]_2_ or in a control mouse treated with PBS. (c) Kaplan-Meier analysis of the survival of mice with intraperitoneal tumors treated with ^213^Bi-DTPA-[F3]_2_ (red), ^213^Bi-DTPA (green) or PBS (black). (d) Macroscopic picture of small intestine and colon of tumor bearing mice treated like those of the tumor prevention study with ^213^Bi-DTPA-[F3]_2_ and PBS (control) 50 days after inoculation with MDA-MB-435 cells i.p.

To study anti-tumor activity of ^213^Bi-DTPA-[F3]_2_, 10^7^ MDA-MB-435 cells expressing firefly luciferase were injected i.p. into SCID-mice. In one set of experiments (tumor prevention study) we tested whether ^213^Bi-DTPA-[F3]_2_ treatment blocks the growth of small intra-peritoneal tumors. Between the days 4–14 after inoculation of tumor cells 6×1.85 MBq of ^213^Bi-DTPA-[F3]_2_ were injected for treatment every other day. In control groups we injected either 6×1.85 MBq of ^213^Bi-DTPA or 6×100 µl PBS. The injection schedule is depicted in figure 4a. Optical imaging revealed that treatment of mice with ^213^Bi-DTPA-[F3]_2_ inhibited tumor growth (Figure 4b). The survival time of mice in each group was then determined. The mean survival of mice in the tumor prevention study treated was 51 and 53 days for mice treated with ^213^Bi-DTPA or PBS, whereas mice treated with ^213^Bi-DTPA-[F3]_2_ lived an average of 93.5 days, indicating a significant (80%; p<0.001) increase of survival by ^213^Bi-DTPA-[F3]_2_ (Figure 4c). We also surgically removed the intraperitoneal tumors in mice treated with ^213^Bi-DTPA-[F3]_2_ or treated with PBS for macroscopic visual examination. As shown in figure 4d 50 days after injection of tumor cells mice treated with ^213^Bi-DTPA-[F3]_2_ exhibited only a small number of tumor nodules whereas mice mock-treated with PBS showed multiple tumors within the peritoneum.

**Figure 5:**
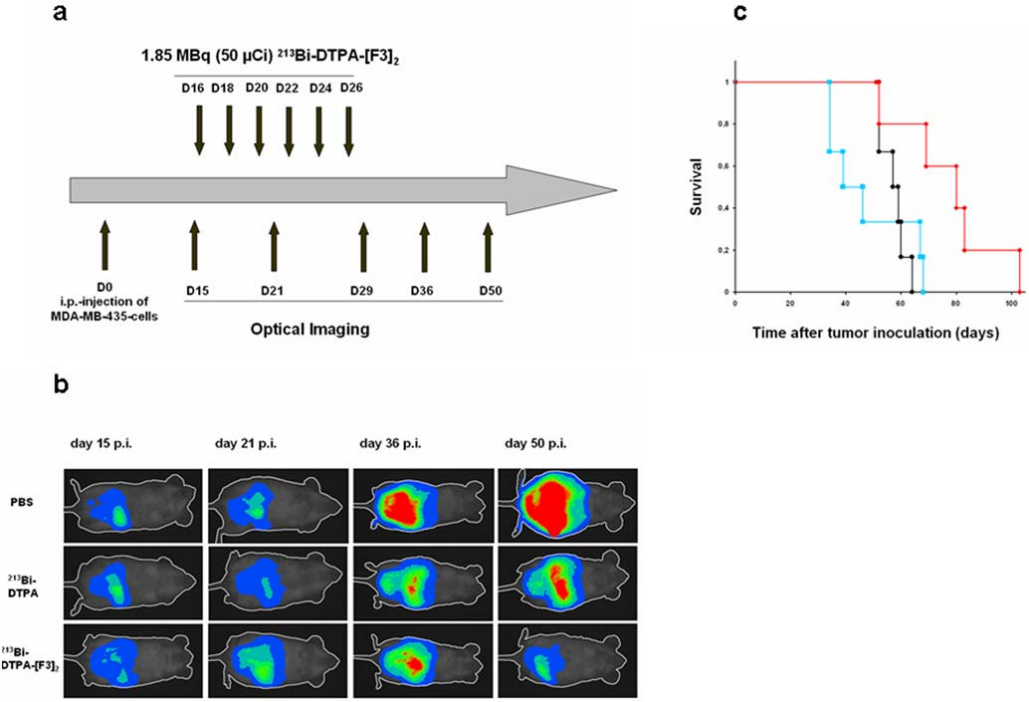
Tumor reduction study. (a) The scheme depicts the time points when ^213^Bi-DTPA-[F3]_2_ was injected and the time points when optical imaging was performed. (b) Assessment of the tumor growth by optical imaging in mice treated with ^213^Bi-DTPA-[F3]_2_ or in control mice treated with PBS or ^213^Bi-DTPA. (c) Kaplan-Meier analysis of the survival of mice with intra-peritoneal tumors treated with ^213^Bi-DTPA-[F3]_2_ (red), ^213^Bi-DTPA (green) or PBS (black).

In a tumor reduction study we tested whether ^213^Bi-DTPA-[F3]_2_ is also effective in the treatment of large intraperitoneal tumors. Between the days 16 and 26 after inoculation of tumor cells we i.p. injected 6×1.85 MBq ^213^Bi-DTPA-[F3]_2_, 6×1.85 MBq ^213^Bi-DTPA or 6×100 µl PBS were (Figure 5a). Again, optical imaging confirmed lower tumor loads after treatment with ^213^Bi-DTPA-[F3]_2_ (Figure 5b). In this study the mean survival of mice was 57 days in the PBS group, 48 days in the group treated with ^213^Bi-DTPA and 78 days in the ^213^Bi-DTPA-[F3]_2_-group, i.e. there was a trend of increased survival which was statistically significant (p = 0.04; Figure 5c).

### Toxicity of ^213^Bi-DTPA-[F3]_2_ -treatment

Our biodistribution study showed that ^213^Bi-DTPA-[F3]_2_ after i. p. injection accumulates in tumors and is excreted via the kidneys. Therefore, primarily renal toxic side effects are to be expected. The serum creatinine level is a highly specific marker of renal function. The serum concentration of creatinine was determined in all tumor bearing mice when they were sacrificed after therapy. We did not observe any significant elevation of the serum creatinine level after treatment with ^213^Bi-DTPA-[F3]_2_ (data not shown) at time of sacrifice.

## Discussion

α-particle emitting isotopes such as ^213^Bi are promising tools for cancer therapy. Due to the high toxicity of α-particles targeted delivery into tumor cells is a prerequisite of their application in oncology. Therefore, carriers that accumulate in tumor tissue but not in other organs are required. Ideally, the unbound molecules should be rapidly excreted and the carrier should mediate an internalization of the nuclide into the nucleus of tumor cells to enhance the DNA-damage and to reduce the cytotoxic effects on neighbouring cells within normal tissue. We generated conjugates of the vascular tumor homing peptide F3 and the metal chelator DTPA which effectively chelate ^213^Bi. ^213^Bi-DTPA-[F3]_2_ is internalized into the cytoplasm and the nucleus of tumor cells, as already reported for FITC-labelled F3-peptide [10]. The peak activity of ^213^Bi-DTPA-[F3]_2_ within the nucleus was found approximately 5 minutes after application but significantly elevated levels were found for at least 90 minutes. As the half-life of ^213^Bi is approximately 46 minutes, a considerable amount of the isotope will decay in the nucleus, damaging the DNA of cancer cells but not the surrounding tissue. Therefore, ^213^Bi-DTPA-[F3]_2_ induces cell death in tumor cells and effectively reduces the number of malignant tumor cell clones *in vitro*. This was demonstrated for of a variety of tumor cell lines from different types of carcinoma including pancreatic-, colon- and ovary carcinoma as well as breast cancer. Within the tumors ^213^Bi-DTPA-[F3]_2_ is primarily located in the tumor periphery and in the peri-vascular region. In contrast, only small amounts of radioactivity were detected in non-target organs except for the kidneys were ^213^Bi-DTPA-[F3]_2_ is present presumably due to secretion of the peptide. In previous studies by Porkka et al. [14] FITC-F3 was also found in single cells in the intestine and in the skin, whereas in our study no relevant activities were found in these tissues. This difference is most likely due to the blood pool present in these organs which masks the quantitatively low yet potentially specific uptake in a small group of cells.

Application of ^213^Bi-DTPA-[F3]_2_ by i.p. injection into animals with peritoneal carcinomatosis of MDA-MB-435 cells blocks tumor growth and increases the survival of the animals. Moreover, ^213^Bi-DTPA-[F3]_2_ not only blocks the growth of small tumor nodules but also reduces the growth of large intraperitoneal tumors.

The internalization of ^213^Bi-DTPA-[F3]_2_ into the nucleus seems to be necessary for its anti tumor activity as ^213^Bi-DTPA had no relevant effect in vivo and was less active in vitro. As F3 is a fragment of the HMGN2 protein which is known to be associated with chromatin in a cell cycle dependent manner F3 transports ^213^Bi potentially into close proximity of DNA enhancing the effect of the α-particles [23]. Taken together, these findings indicate that ^213^Bi-DTPA-[F3]_2_ is a promising new tool in the treatment of peritoneal carcinomatosis which may be helpful in many patients. As for example a high number of patients with ovarian cancer presents with peritoneal carcinomatosis at the time of diagnosis, ^213^Bi-DTPA-[F3]_2_ may be useful in treating small tumor nodules which are not visible by clinical inspection and improve the diagnosis of these patients by preventing tumor growth. ^213^Bi-DTPA-[F3]_2_ may also be useful for palliative treatment in patients with advanced peritoneal carcinomatosis spread throughout the whole abdomen as we found a trend of longer survival of animals treated with ^213^Bi-DTPA-[F3]_2_. As advanced peritoneal carcinomatosis is poorly responsive to chemotherapy and frequently leads to complications such as ascites ^213^Bi-DTPA-[F3]_2_ may also help to alleviate such complications. We found that ^213^Bi-DTPA-[F3]_2_ blocks the clone formation of tumor cells from many tumor types causing peritoneal carcinomatosis, it may be helpful to reduce tumor growth and to reduce complications such as ascites. It has been shown that F3 binds to many tumor entities including breast and prostate cancer suggesting that F3-directed therapy may be widely applicable.

Though α-particles are highly cytotoxic, we did not observe severe side effects in animals i. p. treated with ^213^Bi-DTPA-[F3]_2_ over a three month observation period. We can not exclude the possibility that pathological changes might become apparent at later time points. As biodistribution studies revealed that ^213^Bi-DTPA-[F3]_2_ is excreted via the kidneys but does not accumulate in other organs, primarily renal side effects are to be expected. Assessment of renal function in mice treated with ^213^Bi-DTPA-[F3]_2_ did not show any pathological changes in creatinine levels. As it has been shown that the internalization of F3 into the nucleus is correlated to cell proliferation, in kidney cells nuclear internalization of F3 may be low due to a low proliferation rate resulting in a low nephro-toxicity of ^213^Bi-DTPA-[F3]_2_ [14], [15].

It has been shown that α-emitting isotopes such as ^225^Ac and ^213^Bi coupled to cancer-specific antibodies are effective in the treatment of solid tumors as well as peritoneal carcinomatosis in pre-clinical models [24], [25], [26], [27], [28]. Here we report for the first time on a tumor-homing-peptide as a carrier for the α-particle emitting isotope ^213^Bi. Compared to antibodies peptides may have some advantages For example, an advantage of peptides is the rapid renal excretion within hours. In contrast antibodies show circulation times of up to a week. Therefore radioactively labeled antibodies not bound to their target molecules on tumor cells will lead to higher radiation exposure of the reminder body compared to radioactive labeled peptides, when labeled to α-emitters with long half lifes such as ^225^Ac. As we reported recently that the somatostatin receptor binding peptide DOTATOC coupled to ^225^Ac is more effective in the therapy of neuroendocrine tumors than DOTATOC coupled to ß-particle emitting isotopes [9]. Therefore, we propose that specific tumor-homing-peptides such as F3 will emerge as carriers for α-particle emitting isotopes in the future.
